# Knee–Hip–Spine Syndrome: Improvement in Preoperative Abnormal Posture following Total Knee Arthroplasty

**DOI:** 10.1155/2019/8484938

**Published:** 2019-07-01

**Authors:** Yasushi Oshima, Nobuyoshi Watanabe, Norishige Iizawa, Tokifumi Majima, Mitsuhiro Kawata, Shinro Takai

**Affiliations:** ^1^Department of Orthopaedic Surgery, Nippon Medical School, 1-1-5 Sendagi, Bunkyo-ku, Tokyo 113-8603, Japan; ^2^Department of Orthopaedic Surgery, Kyoto Kujo Hospital, 10 Karahashi Rajomoncho, Minami-ku, Kyoto 601-8453, Japan; ^3^School of Health Sciences, Bukkyo University, 7 Higashitoganoo-cho, Nakagyo-ku, Kyoto 604-8418, Japan

## Abstract

An ergonomic upright body posture is maintained by the alignment of the spine, pelvis, and lower extremities, and the muscle strength of body trunk and lower extremities. The posture varies with age because of the degenerative changes in the involved structures and the weakening of the muscles. The compensatory mechanisms underlying these changes have recently been evaluated, and the loss of lumbar lordosis results in spinal kyphosis, pelvic retroversion, hip extension, knee flexion, and ankle dorsiflexion. These mechanisms are referred to as the hip–spine and knee–spine syndromes. The spine, hip, and knee are anatomically connected, and the pain and discomfort of the lower back, hip, and knee frequently arise due to degenerative changes of these structures. Thus, these mechanisms are considered as the knee–hip–spine syndrome. Spinal fusion, total hip arthroplasty, and total knee arthroplasty are the surgical procedures for severe degeneration, and their clinical outcomes for the affected sites are promising. However, despite surgeries, other structures may degenerate and result in complications, such as proximal junctional kyphosis and hip dislocation, following spinal fusion. Therefore, it is necessary to evaluate each patient under specific conditions and to treat each section while considering associations between the target structure and entire body. The purpose of this article is to introduce postural maintenance, variations with age, and improvements with surgical interventions of spine, hip, and knee as the knee–hip–spine syndrome.

## 1. Introduction

An ergonomic upright body posture is maintained by the alignment of the spine, pelvis, and lower extremities with the support of the muscles of the body trunk and lower extremities, and this posture varies with age because of degeneration of the involved structures and weakening of the muscles. In the advent of an aging society, abnormal posture with body balance disorders is a serious issue because it leads to decline in activities of daily living and health-related quality of life (QOL).

In the last few decades, treatments involving surgical procedures have been developed as specialized interventions for adult spinal deformity (ASD), osteoarthritis of the hip (hip OA), and osteoarthritis of the knee (knee OA). More recently, mechanisms underlying interactions among the spine, hip, and knee as well as compensatory mechanism underlying their deformities have been elucidated. In addition, similar medical conditions have been speculated to arise from different segments. For instance, spinal deformity results in spinal kyphosis; however, progressions of hip and knee OA may lead to the abnormal global sagittal alignment of the body.

Therefore, the purpose of this article is to summarize the current understanding of associations of the spine, pelvis, hip, and knee with the global and abnormal body postures and to explain postural improvements with surgical interventions, including our clinical outcomes of postural variations following total knee arthroplasty (TKA).

## 2. Body Posture

In 1994, Dubousset put forth the concept of “the cone of economy,” which refers to the upright body posture maintained by the skeletal muscle strength of the body trunk and lower extremities and the alignment of the skeletal structures, including the head, spine, pelvis, and lower extremities. Body posture has been considered the chain of balance, with the body being balanced as an inverted pendulum in the standing position such that both feet on the floor act as the fulcrum connecting the ankles, knees, hips, pelvis, spinal segments, and head. The body sways to maintain balance while standing as the shape of a cone, and the body posture remains stable with minimal energy expenditure when the cone size is narrow. In contrast, the body becomes unstable and the energy expenditure increases when the cone size is large as the body trunk is positioned peripherally of the cone. Thus, when the trunk extends beyond cone, supportive devices, such as cane or crutch, are necessary to prevent fall [1].

## 3. Global Alignment

Body alignment is an important index of body balance in static and dynamic conditions, and it is known to vary with age. The ideal alignment of the body is considered to match C7 plumb line, which is the center of the gravity of the body trunk, and the center of the sacral vertical line in the coronal plane. C7 plumb line passes thought the posterior of the rotational center of the hip in the sagittal plane [2, 3]. In the previous study, the global sagittal alignment of 40-year-old healthy individuals was extensively investigated using a three-dimensional X-ray device [4]; the authors reported that the head was almost on the gravity line, as determined using a force plate as a vertical line from the center of gravity. Moreover, the cervical, thoracic, and lumbar regions of the spine showed lordosis, kyphosis, and lordosis in the normal adult posture, respectively. T7 was the posterior apex of the spine and was 5.0 cm posterior to the gravity line, while L4 was 0.6 mm anterior to the gravity line. The hip center was 1.4 cm anterior to the gravity line, whereas the knee and ankle centers were 2.4 mm and 4.8 cm posterior to the gravity line, respectively. When the ideal alignment of these segments is disrupted due to degenerative change of the involving structures, the underlying compensatory mechanisms preserve the body balance. Otherwise, the body becomes instable and loses balance.

## 4. Fall

The frequency of fall increases with age. Approximately 30% of the individuals older than 65 years of age fall more than once a year. Falls were reported to account for 10% of the emergency room visits and 6% of the hospital admissions [5].

Compared with those who did not fall, individuals who suffered falls showed reportedly poorer body balance, spinal sagittal alignment, muscle strength, and gait speed [6]. Falls in older people, especially in those with osteoporosis, may cause fractures, including vertebral compression as well as proximal femoral and distal radius fractures. Majority of these fractures can easily occur as low-energy injuries with fall from standing height. In older individuals who suffered proximal femoral fracture, 1-year incidence of second hip contralateral fractures was 2.7%–9% [7, 8] and 1-year mortality was approximately 10% [9].

Therefore, preventing falls via strategies involving exercises is crucial considering the higher overall 30-day mortality rate of older patients than that of younger patients following bone fractures [10, 11].

## 5. Sagittal Vertical Axis (SVA) and Sagittal Malalignment

On the contrary to coronal alignment, global sagittal alignment and pelvic version are associated with health-related QOL in terms of pain and disability [12]. Thus, many studies have focused on global sagittal alignment.

SVA, defined as the horizontal distance from the posterosuperior corner of S1 to the plumb line dropped from the center of the body at C7, is one of the indices of the sagittal body balance. A positive SVA indicates a plumb line passing anteriorly to the front of the sacrum, while a negative SVA indicates a plumb line passing through or behind the sacrum.

SVA of adolescents is significantly more negative than that of adults [13]. Thereafter, SVA has been reported to increase with age [2, 4]. Higher SVA values indicate forward bending the body trunk, resulting in low back pain, difficulty in touching the top of the shelf, and performing routine tasks, all of which lead to restricted activities of daily living. Consequently, back muscle strength is reduced, and the vertebral spine motion is limited. The vision is directed downwards, which impairs the ability to grasp the circumstances quickly and may lead to imbalance, walking disturbances, and falls [6, 12, 14].

SVA is an index of a stable standing posture, in which the femur is fixed and the gluteus maximus is contracted such that the pelvis is tilted posteriorly [15]. Thus, SVA is an indicator of changes due to the actions of compensatory mechanisms of the pelvis, hip, and knee [16, 17]. However, in the dynamic status, the femur is not fixed and the pelvis may tilt anteriorly [18]. Therefore, additional indices must be applied to dynamic alignment while performing normal activities.

## 6. ASD

Degenerative spinal kyphoscoliosis of the coronal and sagittal alignments occurs with degeneration of the facet joints and/or spinal discs, vertebral compression fractures, and weakening of the lumbodorsal muscles. Reduced lumbar lordosis (LL: the sagittal Cobb angle measured between the superior end plate of L1 and the superior end plate of S1) and excessive spinal kyphosis are associated with increased intradiscal pressure, which may cause low back pain [19]. Approximately 60% of the older individuals are considered to present with ASD; however, some patients with abnormal posture do not show any spinal deformities [20].

According to the Scoliosis Research Society-Schwab Classification, ASD is characterized by coronal curves of 4 types: type T, a thoracic major curve of >30° (apical level of T9 or higher); type L, a lumbar or thoracolumbar major curve of >30° (apical level of T10 or lower); type D, a double-major curve, with each curve >30°; and type N (normal), no coronal curve >30° (i.e., no major coronal deformity) [20]. Moreover, sagittal alignment is characterized through 3 modifiers: (1) the difference between the angle of the pelvic incidence (PI: the angle between the line drawn perpendicular to the sacral end plate at its midpoint and the line drawn from the midpoint of the sacral end plate to the midpoint of the bicoxofemoral axis) and LL; (2) the pelvic tilt (PT: the angle between the line connecting the midpoint of the sacral end plate to the midpoint of the bicoxofemoral axis and the vertical); and (3) SVA (SVA > 40 mm indicates poor sagittal alignment).

Variations in the spinal alignment are related to the pelvis position as a compensatory mechanism. When LL increases, the pelvis tilts anteriorly and thoracic kyphosis (TK) increase as a reciprocal change. In contrast when LL decreases, the pelvis tilts posteriorly and TK increase and the knee flexes. However, when this mechanism cannot maintain body posture, the thoracic and lumbar kyphosis increase, the body bends forward, and the gravity line moves forward, giving rise to severe abnormal posture with the stretching of the erector spinae muscles and chronic low back pain [2].

The pelvis has a fundamental role as the main regulator of the chain of correlation between the spine and lower extremities in this compensatory mechanism. Thus, the parameters LL, PT, sacral slope (SS; the tangent line to the superior endplate of S1 and the horizontal plane), and PI (the sum of PT and SS, which does not change with patient position, activity, age, or structural deformity) are important in the evaluation of the relationship between the spine and pelvis [12, 21].

## 7. Hip and Knee OA

OA is the most common form of arthritis and involves inflammation and major structural changes of the joints, causing pain and functional disabilities.

The risk of knee OA increases with age, with the global prevalence of radiographically confirmed symptomatic knee OA estimated to be 3.8%. In contrast, the incidence of hip OA is less than that of knee OA, with the global age-standardized prevalence of symptomatic radiographically confirming hip OA being 0.85% [22].

Coxitis knee is defined as the secondary knee OA, which occurs due to hip OA. Originally, coxitis knee was believed to be related to hip disease. However, adduction contracture of the hip and discrepancies of leg length have been regarded as the main etiologies of coxitis knee [23]. Of note, although an important symptom of hip and knee OA, coxitis knee affects the coronal alignment of the body posture to a greater extent rather than the sagittal alignment for the body posture.

## 8. Hip–Spine Syndrome

The concept of hip–spine syndrome, in which the spinal and hip diseases are related and/or concomitant, was first proposed in 1983 [24]. This syndrome was classified into 4 groups: simple, complex, secondary, or misdiagnosed hip–spine syndrome.

Subsequently, the association between spinal alignment and hip was studied. Nonetheless, it is necessary to evaluate hip pathology and spinal alignment in hip OA treatment [25].

In the coronal plane, spinal scoliosis shifts the center of the body gravity line, and the mechanical load increases on one side of the hip and results in hip pain of the affected side. In this situation, the compensatory mechanism of the pelvis is activated, with the gluteus maximus being responsible for the reciprocal movement of the spine and pelvis.

On the other hand, the sagittal alignment progresses to spinal kyphosis and pelvic retroversion with age. In such cases, the loading area of the acetabulum reduces and the load per unit area increases significantly; this is considered to be the mechanism of the progression of primary hip OA. When the hip develops a fixed flexion deformity, there may be an associated loss of LL. Thus, in patients with severe hip OA, the body trunk bends forward, resulting in low back pain and sagittal malalignment. In contrast, in acetabular dysplasia of the hip, which is one of the main causes of secondary hip OA, the front coverage area of the acetabulum remains anatomically small. Thus, the compensatory mechanism activates pelvic anteversion, leading to increased LL and SS [25] and eventually resulting in vertebral disk dislocation, foraminal stenosis, radiculopathy, and low back pain [26]. In this situation, the compensatory mechanism is not activated when the spine too undergoes degenerative changes and the hip OA may thus progress.

Since symptoms of lumbar radiculopathy of L3 root nerve greatly vary from thigh pain to hip and/or knee pain, they may appear similar to pain due to hip or knee OA [27]. Thus, lumbar disease might be mistreated as hip and/or knee diseases. Therefore, lumbar diseases should be considered in nonrespondents of hip and knee OA treatment [28].

## 9. Knee–Spine Syndrome

In the sagittal alignment, spinal kyphosis induces pelvic retroversion, hip extension, knee flexion, and ankle dorsiflexion as compensation mechanisms [12]. These mechanisms increase the load on the knee joint and lead the progression of knee OA.

A study evaluating compensatory mechanisms of knee OA in older patients reported that knee flexion in mild knee OA was mainly compensated with the lumbar spine to reduce LL and move C7 plumb line forward [29]. Moreover, sagittal balance was not compensated by the lumbar spine only in severe knee OA; the hip was flexed and the pelvis was anteverted with significant forward spinal inclination, resulting in an unbalanced status.

Spinal compensatory abilities are limited for knee flexion because older patients might have spinal deformity, and the flexibility of the spine may be poor [17, 29]. In this condition, the head goes forward, SVA increases, and sagittal malalignment leads to low back pain. Interestingly, knee flexion contracture can lead to loss of LL and anterior sagittal shift in young individuals without any spinal pathology, although it does not influence the pelvis [17].

Tsuji et al. described a correlation between sacral inclination and patella-femoral pain, which is related to changing of lumbar alignment as the knee–spine syndrome [30]. After that, the relationship between the knee and spine involving pelvis has been examined. Murata et al. also evaluated the correlation between the restriction of knee extension and loss of LL [31]. Lumbar malalignment is common in patients older than 70 years of age; however, such restriction of knee extension is predominantly found in patients older than 60 years of age. Therefore, knee OA may occur first, followed by spinal deformity. Harato et al. suggested knee flexion contracture significantly influenced three-dimensional trunk kinematics and would lead to spinal imbalance [32]. Moreover, Tauchi et al. demonstrated the relationship between increased spinal inclination and knee OA as the knee–spine syndrome [33].

## 10. Spinal Fusion Surgery and Alignment

Loss of LL with age is a common cause of sagittal malalignment of the spine. When the body trunk is located peripherally to the shape of the cone along with markedly increase SVA, surgical interventions with spinal osteotomy and fusion have been applied to improve the abnormal posture. Although the surgical algorithmic approaches for ASD have not been established, a postoperative SVA of <50 mm has been suggested as an optimal target [21, 34]. Aggressive correction of the malignment in older patients increases the risk of proximal junctional kyphosis and treatment failure. However, inflexible deformities generally require more aggressive approaches to achieve adequate correction. Thus, careful planning with operative alignment target and determining spinal flexibility are important to obtain successful outcomes [21].

## 11. THA and Alignment

Implantation of the acetabular cup in the ideal orientation is necessary to reduce edge loading and to avoid articular impingent; the malposition of prostheses in THA accelerates polyethylene wear and increases risk of hip dislocation. Traditionally, the anteroposterior view of the radiography has been applied to evaluate the cup positioning and implantation of the prosthesis within the safe zone. However, patients with normal standing cup orientation occasionally experience dislocation. Therefore, a new idea of the functional orientation of the acetabular cup has been developed recently, wherein the sagittal pelvic kinematics is involved [35, 36].

Pelvic retroversion progresses with age regardless of THA, and the risk of superior edge loading and anterior hip dislocation increases with hip extension. Patients with sagittal deformities are at an increased relative risk of dislocation and THA revision [37]. Moreover, spinal fusion following THA increases the risk of hip dislocation, particularly involving the fusion of the sacrum. Anterior impingement of the hip in deep hip flexion may be the risk factor for posterior THA dislocation while in stand up from the chair because the pelvis is surgically fixed [38–40]. Moreover, THA following spinal fusion is associated with a high risk of hip posterior dislocation because the pelvis is not posteriorly tilted, which may occur even in the sitting position [35].

Therefore, different reactions of the pelvis in individual patients need to be carefully considered during THA to prevent postoperative hip dislocation. More studies regarding cup positioning are necessary; however, restoration of normal sagittal balance and consideration of patient-specific pelvic kinematics are speculated to be critical [14, 36].

## 12. TKA and the Alignment

Muscle weakness of the lower extremities and restriction of knee extension in knee OA have been considered to be associated with loss of LL and abnormal posture [31]. However, malalignment of the lower extremities and limited range of motion of the knee, particularly extension, can be improved by TKA. Thus, effects of the spinopelvic alignment following TKA were evaluated, and SS was reported to increase postoperatively in the preoperative knee contracture of >10° [41].

Recently, effects of TKA on sagittal malalignment and variations in the alignment of the spine, pelvis, and lower extremity following TKA were assessed prospectively in our institution [42]. In the present study, the hip flexion was defined as the angle between the femoral shaft and vertical line in the standing position, and SVA ≤ 40 mm was considered normal. Patients with primary knee OA, who were scheduled to undergo primary TKA, were enrolled. However, patients with arthritis secondary to another etiology, such as rheumatoid arthritis, trauma, and previous surgical interventions to the knee, were excluded. Moreover, patients with hip and ankle OA, cranial nerve diseases, and severe spinal deformity were excluded. The study was approved by an institutional review board, and informed consent for participation was obtained from all patients. We observed different patterns of postural changes as well as hip and knee angles following TKA. Three typical cases are presented.

**Figure 1:**
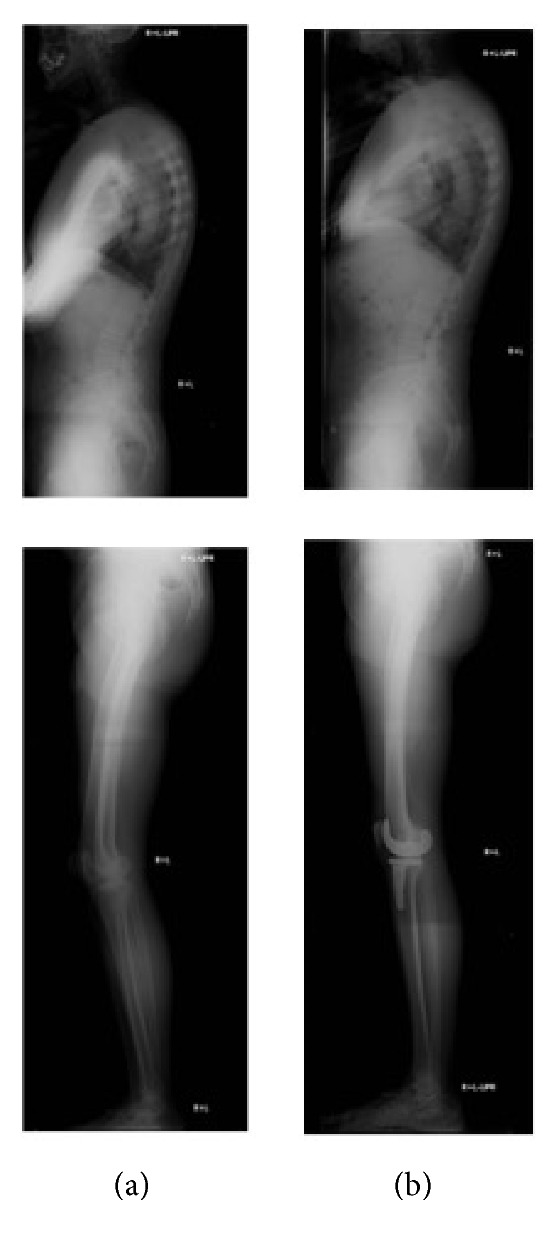
A case of normal SVA with the extension of knee joint (a) before and (b) after the surgery.

*Maintained Normal SVA with the Extension of the Hip and Knee Joints*. A case of 69-year-old man who underwent right TKA: the preoperative roentgenographic parameters of SVA, TK, LL, PI, PT, SS, hip flexion, and knee flexion were 14.8 mm, 34°, 40°, 52°, 20°, 32°, 7°, and 18°, respectively (Figure 1(a)). Preoperative SVA was normal and hip and knee joints were flexed while standing. At 12 months after TKA, the parameters of SVA, TK, LL, PI, PT, SS, hip flexion, and knee flexion became 0 mm, 35°, 45°, 55°, 23°, 32°, 1°, and 8°, respectively (Figure 1(b)). These results indicated that SVA remained normal with the extension of hip and knee joints. Some of the patients with the preoperative normal SVA showed that the hip and knee positions tended to extend, lumbar lordosis was decreased, and SVA was increased; however, SVA was still almost within the normal range after TKA.

**Figure 2:**
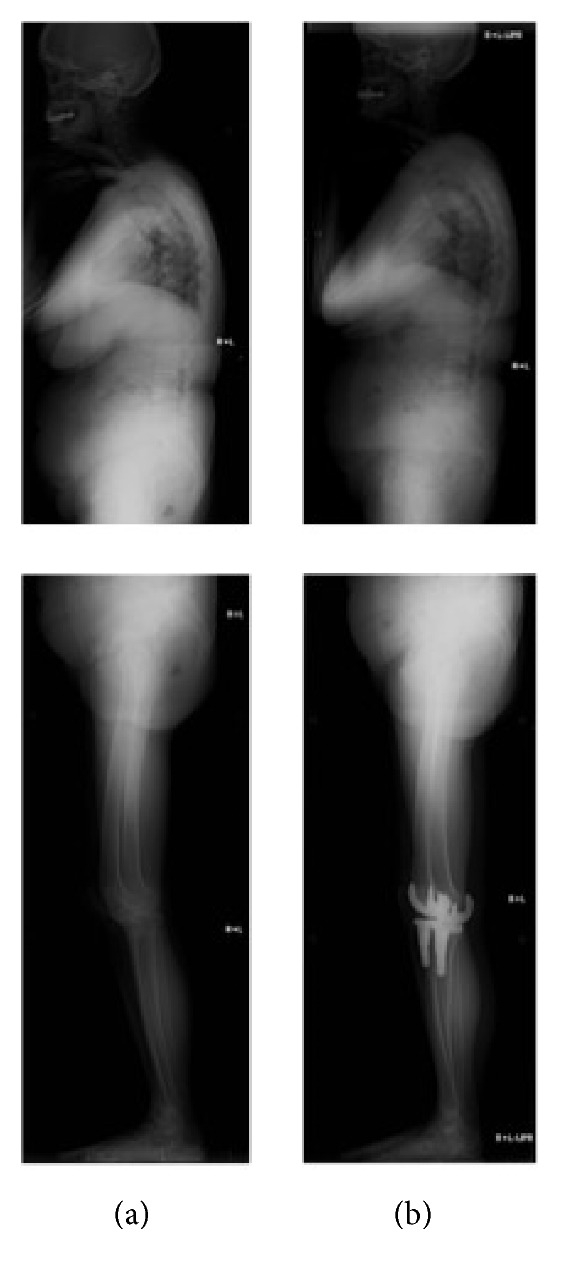
A case of improved abnormal SVA with the extension of knee joint (a) before and (b) after the surgery.

*Abnormal SVA in spite of the Extension of the Hip and Knee Joints*. A case of 72-year-old woman who underwent bilateral TKA as a two-stage surgery: the preoperative parameters of SVA, TK, LL, PI, PT, SS, hip flexion, and knee flexion were 75.0 mm, 23°, 23°, 58°, 35°, 23°, 6°, and 17°, respectively (Figure 2(a)). Postoperative evaluations were performed after 19 and 6 months of right and left TKA, respectively. Postoperative parameters of SVA, TK, LL, PI, PT, SS, hip flexion, and knee flexion became 49.7 mm, 21°, 25°, 54°, 35°, 19°, 5°, and 10°, respectively (Figure 2(b)). Some of the patients with the preoperative abnormal SVA showed that the hip and knee tended to extend; however, pelvic posterior tilt was not improved and SVA was increased, or PT and SVA were increased.

**Figure 3:**
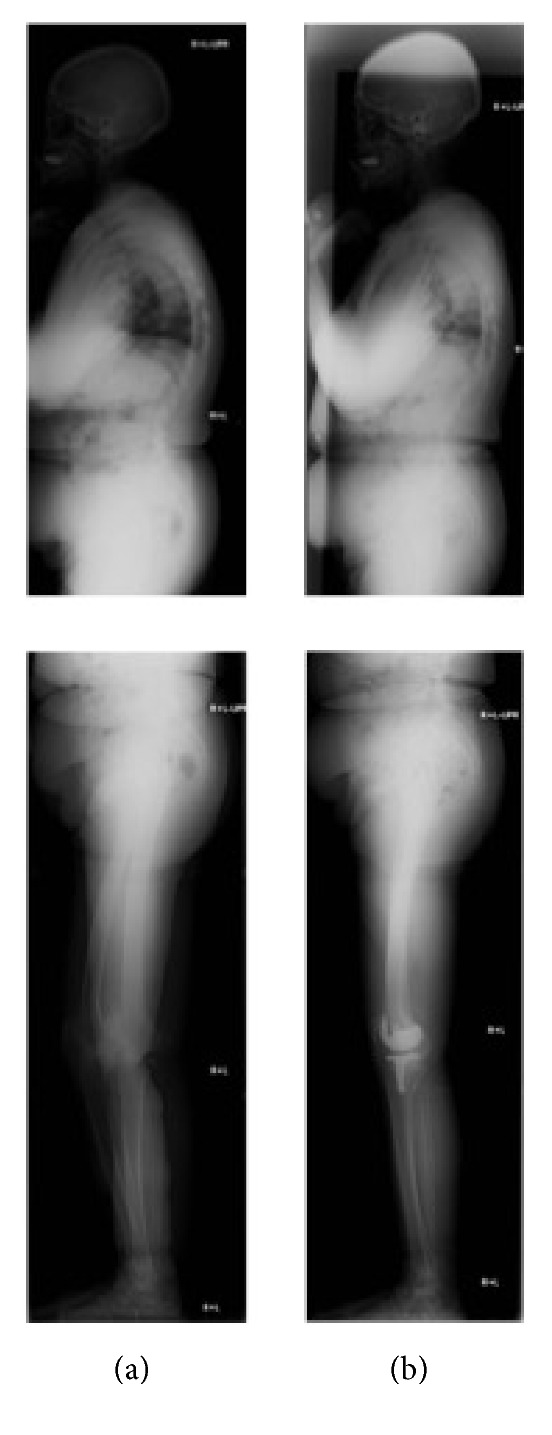
A case of the improved SVA with the extension of hip and knee joints (a) before and (b) after the surgery.

*Decreased SVA with the Extension of the Hip and Knee Joints*. A case of 74-year-old woman who underwent left TKA: preoperative parameters of SVA, TK, LL, PI, PT, SS, hip flexion, and knee flexion were 70.5 mm, 58°, 57°, 58°, 25°, 33°, 8°, and 17°, respectively (Figure 3(a)). At 12 months after TKA, the parameters of SVA, TK, LL, PI, PT, SS, hip flexion, and knee flexion became 28.4 mm, 50°, 55°, 57°, 23°, 34, 4°, and 7°, respectively (Figure 3(b)). Thus, most patients with the preoperative abnormal SVA showed that the hip and knee became extended, lumbar lordosis increased, and pelvic posterior tilt decreased even after the short-time period of TKA.

**Figure 4:**
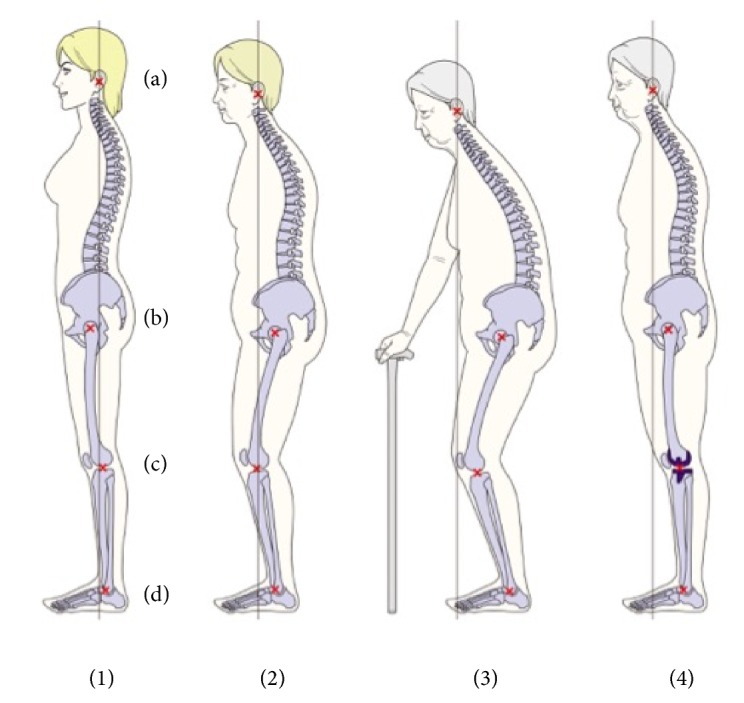
Age-related global postural changes. (a) The center of the acoustic meati, (b) the center of the hip, (c) the center of the knee, and (d) the center of the ankle. The vertical line shows the plumb line from the center of the acoustic meati. (1) The global alignment of the healthy subject. The plumb line from the center of the acoustic meati is close to the gravity line. The cervical and thoracic vertebrae are posterior to the gravity line. The lumbar vertebrae show lordosis, and L4 is anterior to the gravity line. The sacrum is posterior, and the hip center is anterior to the gravity line. The knee joint and ankle joint are posterior to the gravity line. (2) TK increases, LL decreases, and the pelvis tilts posteriorly while the hip, knee, and ankle flex. Consequently, the sagittal balance sifts anteriorly with age. (3) Older subjects show spinal kyphosis with the severe anterior shift of the sagittal balance. Consequently, the body balance is better maintained with support. (4) As the knee becomes extended and the lower extremity alignment is corrected with TKA, the global alignment and the sagittal balance can be improved.

Together, these data suggest that knee OA results in the knee flexion while standing. With the progress of knee OA, hip is flexed, pelvis is posteriorly tilted, and the spine is bent forward. However, once the restriction of knee extension is corrected and lower extremity alignment is improved following TKA, lumbar and pelvic parameters are affected even in patients with normal SVA before surgery. Moreover, preoperative abnormal SVA tends to decrease with changes in knee flexion and lumbar parameters as knee–hip–spine syndrome (Figure 4). Therefore, TKA may help recovered knee function and correct lower extremity alignment in severe knee OA as well as improving posture to prevent falls.

## 13. Knee–Hip–Spine Syndrome

The current THA and TKA strategies have been developing since 1960s. At that time, the longevities of the artificial prostheses were much shorter than those in recent years. However, there were surgeries indicated for older patients and the mean lifespan was much shorter than the present years. Thus, the surgeons only needed a short observation period of the affected site.

Recently, the longevities of prostheses and surgical procedures have dramatically improved. These surgeries have also been performed in younger patients, and generally the mean lifespan has become longer than before. Therefore, long-term follow-up is indispensable after the primary surgery. During this longer period, additional concomitant symptoms may occur with age followed by the original pathology. In addition to THA and TKA, reconstruction of the spinal alignment with the spinal long fusion has emerged as a new approach. Therefore, long-term follow-up after surgeries as well as additional treatments is essential for older patients with complex deformities of multiple structures to improve their QOL.

The spine, hip, and knee are anatomically connected, and all these structures undergo degenerative changes with age as ASD, hip OA, and knee OA. Clinically, pain and discomfort of the low back, hip, and knee are commonly occurring simultaneously, which are referred to as the knee–hip–spine syndrome. Occasionally, physicians misdiagnose and treat the wrong structures. The pendulum test is recommended to detect hip pathology from these symptoms to prevent misdiagnosis [43]. In addition, the abnormal posture occurs with ASD, hip OA, and knee OA. Treatments for the spine, hip, and knee are separated and specialized; however, physicians often carefully examine patients under the consideration with the knee–hip–spine syndrome as differential diagnosis.

At present, physicians often face new challenges of treating the complex multiple degenerative structures or additional degenerative changes following the changes to the primary affected structure. Thus, it is necessary to understand the knee–hip–spine syndrome and to elucidate the etiologies and compensatory mechanisms of the spine, hip, and knee, individually.

## 14. Conclusion

Musculoskeletal structures are interconnected, and pathologies of these structures are collectively referred to as the knee–hip–spine syndrome. Thus, when each structure is treated, its effect on the entire body must be considered. In this article, we reviewed the association between knee, hip, and spine, and our findings may be helpful for orthopaedic surgeons to bridge the gap between the treatments of these structures.

## Data Availability

Aggregate data are included in the results section. Please contact the corresponding author for raw data, including individual ratings.

## Abbreviations

QOL: Quality of life
ASD: Adult spinal deformity
hip OA: Osteoarthritis of the hip
THA: Total hip arthroplasty
knee OA: Osteoarthritis of the knee
TKA: Total knee arthroplasty
SVA: Sagittal vertical axis
TK: Thoracic kyphosis
LL: Lumbar lordosis
PI: Pelvic incidence
PT: Pelvic tilt
SS: Sacral slope.
